# The College of Medicine in the Republic of Malawi: towards sustainable staff development

**DOI:** 10.1186/1478-4491-5-10

**Published:** 2007-04-13

**Authors:** Ed E Zijlstra, Robert L Broadhead

**Affiliations:** 1 College of Medicine, PO Box 360, Blantyre, Republic of Malawi

## Abstract

**Background:**

Malawi has a critical human resources problem particularly in the health sector. There is a severe shortage of doctors; there are only few medical specialists. The College of Medicine (COM) is the only medical school and was founded in 1991. For senior staff it heavily depends on expatriates. In 2004 the COM started its own postgraduate training programme (Master of Medicine) in the clinical specialties.

**Methods:**

We explore to what extent a brain drain took place among the COM graduates by investigating their professional development and geographical distribution. Using current experience with the postgraduate programme, we estimate at what point all senior academic positions in the clinical departments could be filled by Malawians. We demonstrate the need for expatriate staff for its most senior academic positions in the interim period and how this can be phased out. Lastly we reflect on measures that may influence the retention of Malawian doctors.

**Results:**

Since the start of the COM 254 students have graduated with an average of 17 students per year. Most (60%) are working in Malawi. Of those working abroad, 60% are in various postgraduate training programmes.

In 2015, adequate numbers of Malawi senior academics should be available to fill most senior positions in the clinical departments, taking into account a 65% increase in staff to cope with increasing numbers of students.

**Conclusion:**

There seems to be no significant brain drain among graduates of the COM. The postgraduate programme is in place to train graduates to become senior academic staff. In the interim, the COM depends heavily upon expatriate input for its most senior academic positions. This will be necessary at least until 2015 when sufficient numbers of well trained and experienced Malawian specialists may be expected to be available. Improved pay structure and career development perspectives will be essential to consolidate the trend that most doctors will remain in the country.

## Background

Malawi is among the poorest countries world-wide with a gross domestic product (GDP) of US$ 519 per capita [1]. It has a huge human resources problem, particularly in the health sector. Malawi ranks last on the WHO list of estimates of health personnel with 2 doctors per 100 000 people [1]. There was no Malawi medical school before the College of Medicine (COM) was established in 1991. Before that students were sent to medical schools in neighbouring countries and later abroad, in particular to the United Kingdom of Great Britain and Northern Ireland (U.K.) and the United States of America. Many graduates did not return and it was felt that the medical training received abroad was not appropriate for a doctor working in an African setting [2]. The curriculum at the COM was introduced in a gradual manner, and in 1998 the first students fully trained in Malawi graduated. The curriculum was based on the traditional British format and reviewed by external consultants in several curriculum conferences. It is a 5-year programme that leads to a Medical Bachelor and Bachelor of Surgery (MBBS) degree. After another 18 months of internship the doctor can be registered with the Medical Council of Malawi. In 2004 the COM introduced its own postgraduate programme as a 4 year Master of Medicine (M. Med) degree programme which qualifies the candidate for registration as a specialist.

The loss of health professionals from developing countries is widely recognized as a threat to the solution of the human resources crisis in the health sector of developing countries especially in Africa [3]. It was estimated that 60% and 70% of health-care workers left Ghana in the 1980s and Zimbabwe in the 1990s respectively [4]. In particular the UK played a role in this migration because its National Health Service (NHS) heavily depends on expatriate doctors. Unsurprisingly, it has a higher proportion of doctors trained overseas than any other country (UK > 31%; France, Germany ≤5%) [3]. Over 5000 doctors from sub-Saharan Africa have migrated to the USA, mainly from Ghana, South Africa and Nigeria [5]. Clearly the retention of Malawian doctors in Malawi is of utmost importance not only for the country as a whole but in particular for the CoM that heavily depends on expatriate doctors and specialists for its academic staff. The clinical departments typically have an establishment of 8 positions of which on average 1 (range 0 – 4) is filled by a Malawian at the Senior Lecturer level. All positions at the level of Associate Professor or Professor are filled by expatriates as the pool of Malawian specialists is small and those comprising it are still in the early stages of their academic careers. Of the eight positions in the establishment, it is usually not possible to attract more than four specialists, leaving the other four vacant. These are usually filled by younger expatriate doctors who are still in training themselves but who function as lecturers in the College.

In this paper we describe the professional development and geographical distribution of the COM graduates after the first 15 years. We describe projected staff requirements with special reference to inflow of Malawian senior staff and decreasing dependence on expatriates. Lastly, we explore measures that may be effective to retain the graduates in the country while at the same time assuring the highest possible level of training and career opportunities.

## Methods

We have documented the professional development and geographical distribution of all the COM graduates by contacting them directly or through their peers. This process took place in September 2006.

Based on numbers of students expected to graduate, the need for more staff as numbers of students increase and the current recruitment rate of the postgraduate programme, we attempted to estimate when a typical clinical department would have senior staff consisting only of Malawians. We attempted to quantify the dependency on expatriate staff in the interim period and how this can be phased out.

## Results

### College graduates

**Figure 1:**
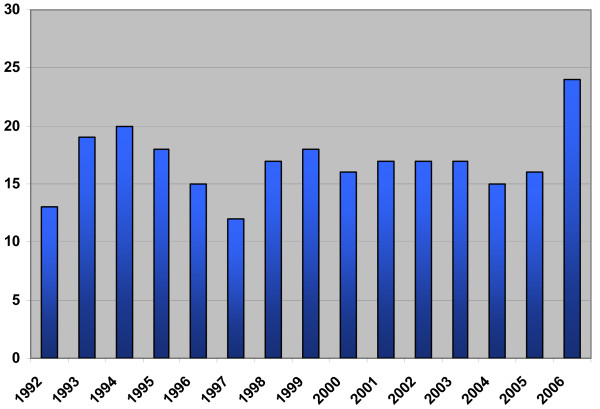
Number of graduates from the College of Medicine since its establishment in 1991.

254 students have graduated since 1991 with an average of 17 per year (Figure 1). Of those who graduated, 76% are male and 24% are female.

Eight graduates have died.

**Figure 2:**
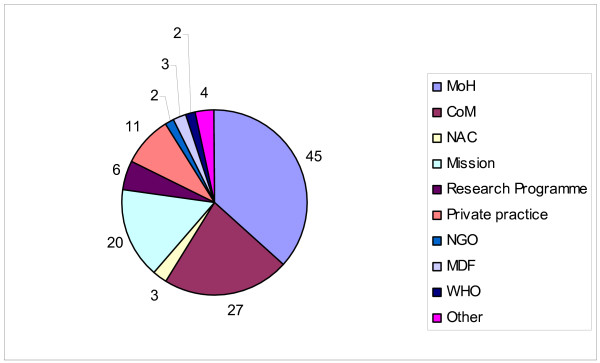
**Type of organization in which COM graduates are employed and who are in Malawi (n = 123)**. MoH Ministry of Health. CoM College of Medicine. NAC National AIDS Commission. NGO Non Governmental Organization. MDF Malawi Defence Force. WHO World Health Organization.

We were not able to collect information on professional development for 7 graduates (3%); 6 of those left Malawi, for 1 the country of residence is unknown. Of 206 graduates who are registered as medical practitioners, 60% are working in Malawi (Figure 2); 48 (39%) work for the Government with 4 in executive positions. The majority are contributing to health care in Malawi in the public sector. Only 9% of graduates are mainly involved in private practice. Nineteen graduates have now qualified as specialists and 78 are working as medical officers or District Health Officers.

There are 83 (40%) graduates currently working abroad, of these 48% are in the UK. It is unlikely that at least 24 will return, for reasons of marriage, gross overstay of the training period, or permanent appointment as a consultant. Forty-nine graduates are in various postgraduate training programmes.

### Staff projection until 2015

**Figure 3:**
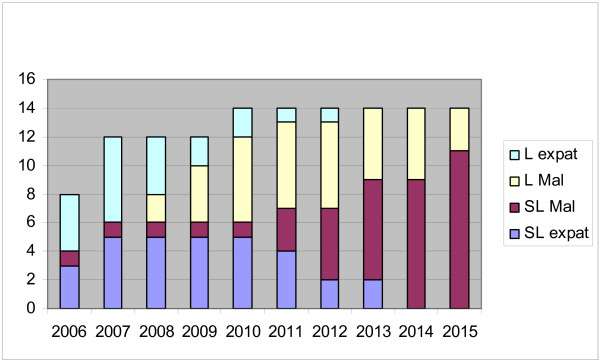
**Staff development in the Department of Medicine until 2015**. L expat Expatriate at lecturer level. L Mal Malawian at lecturer level. SL Mal Malawian at senior lecturer level or above ([associate] professor). SL expat Expatriate at senior lecturer level or above ([associate] professor).

Figure 3 shows the projected staff development until 2015 taking the Department of Medicine as an example. Several assumptions were made: in order to effectively run a teaching programme in a department, a minimum of four senior staff at the level of Senior Lecturer, Associate Professor, or Professor are needed at any point in time. In addition, as the numbers of students are increasing, more staff are needed. Using a simple questionnaire, all departments were asked to estimate the number of staff at various levels needed to cope with the increasing number of students. In general an increase of 65% was felt to be necessary. It is probably not realistic to expect more than three postgraduate students to enter the M.Med per year and that two of those will remain in the COM. For all four clinical departments combined it means that in the interim the number of senior expatriate staff needed is twenty from 2007–2010, while decreasing to sixteen and eight in 2011 and 2012, respectively.

## Discussion

The number of graduates of the COM has been relatively small but is expected to increase as the intake in year one has increased from 30 to 60 in 2001 and should increase to 100 in 2010. Given the current numbers of undergraduate students, the gender balance is expected to improve from 24% to 35% females by 2010. As aforementioned, eight graduates have died; similar significant death rates among medical graduates have been reported from Uganda, most of which were thought to be HIV related [6]. The majority of graduates are in Malawi and working in the health sector. Of those (49) who are in various postgraduate training programmes, most are in the UK. In the past these training initiatives were not well structured and were often open-ended, particularly for those who were sent to the UK. The College has decided not to encourage this type of training any longer as many students do not come back and remain in the NHS. The reason that the College encouraged some of its graduates seeking specialist qualifications to go to the UK in the early 90s was because once in work the graduates earned a good salary which spared the College the trouble of finding 'full fellowships'. It also provided good 'hands-on' clinical experience. The recent legislation restricting permanent appointments to EU candidates in preference may have some effect in persuading those not in permanent appointments to return.

In 2004 the College has, with support principally from the Netherlands, Norway and Sweden, started its own postgraduate programme that has the format of a 4 year Master of Medicine degree in Medicine, Paediatrics, Surgery, Obstetrics and Gynaecology, Anaesthesia and Ophthalmology. It encompasses a part I and II (2 years each) and a dissertation on a research project. For all components an external examiner from outside Malawi is invited for quality control. There has been help from the Royal Colleges in the UK in developing this initiative.

In the 'Part I' period of training in Malawi the students work as registrars in their designated department. The COM is affiliated to the Queen Elizabeth Central Hospital (QECH) which has the status of a teaching hospital. Throughout the 2 years there is protected time for formal teaching. After passing the Part I examination, a well defined period is spent in a country with the highest standards in clinical specialties. This is necessary because the current support services at QECH are not of a sufficient standard to give adequate experience. South Africa is the obvious country of choice because it is a SADC neighbour and the patterns of illnesses are similar. South Africa also includes western diseases and it has a high standard in medicine. Importantly, visas for South Africa are only granted for the duration of the training after which it is impossible to stay. The trainee is therefore likely to return to Malawi. All posts are supernumerary and currently funded by the Netherlands. This policy confidently allows the students to have positions as registrars with similar duties and learning opportunities as their South African counterparts, rather than being observers only [7]. The first postgraduate students in Medicine and Paediatrics are now in Johannesburg (University of Witwatersrand) and Durban (University of KwaZulu-Natal), respectively. After returning to Malawi, they will prepare for the Part II examination and write a dissertation on their research project. It is hoped that a number of candidates will pursue an academic career and join the College to become teachers themselves.

In the meantime, the College is short of staff and heavily depends on expatriates to fill its academic positions. This makes it vulnerable and unstable. The current shortage of staff was highlighted in a recent evaluation report by a team from the World Health Organization (WHO) [8]. Donors are often reluctant to provide technical assistance, because of fear that the presence of expatriate doctors would negatively influence the career perspectives of Malawi nationals. This is a serious misconception as the pool of Malawian doctors currently in training is simply too small to produce sufficient experienced academic senior staff. On the contrary, the input of expatriate doctors is essential for the foreseeable future in order to achieve the ultimate goal of an academic staff consisting of Malawians in most if not all senior positions. In addition, as the number of students is expected to increase to 100 per year by 2010, more members of staff are needed.

By mid 2007 the Dutch Government will have supported technical assistance in Malawi for 40 years but has decided to withdraw its funding because of a change in priorities. The COM experienced a similar change of policy when Overseas Development Administration (ODA – now Department for International Development, DfID), which previously supported the creation of the College, withdrew support in 1994. This withdrawal is premature and puts the COM at risk. Having short term specialist input from overseas in teaching has been suggested as an alternative. While this is in itself useful, it is unrealistic to expect these specialists to be away from their practice for more than 2 weeks. In addition, they would not be able to replace long term senior staff members who design, oversee and adjust the curriculum whenever necessary and who participate in the various supervisory academic committees.

Ironically, because of the current human resource crisis in medicine, the United Nations Development Programme (UNDP) are sponsoring doctors as United Nations Volunteers at USD 40,000 per doctor per year to fill gaps that cannot be filled by Malawians. These foreign doctors are often at a disadvantage, especially when practicing in the community because of difference in training, culture and language.

It is difficult to predict when sufficient Malawian senior staff will be available to take over from the expatriate staff. It depends primarily on numbers of candidates who enter the M.Med programme. The pool of candidates is desperately small but is expected to increase as numbers of graduates increase. Nevertheless predictions must be made and we have attempted to quantify the need for expatriate staff during the transition period.

In the interim, because of the increasing numbers of students, the demand for expatriate senior and junior staff increases initially before it gradually phases out in 2015. Obviously this may be achieved earlier or later, depending on Malawian staff already in place and the number of postgraduate trainees joining the COM.

### The way forward

Clearly it will take many years before well trained and experienced Malawians can compete for the senior positions which will create stability in teaching and management.

The policy of a bold increase in number of students 5 years ago is beginning to bear fruit and the pool of Malawian doctors is steadily increasing. A further increase is planned around 2010. There is now an attractive M.Med programme of which the College is in full control. However, it is still in its infancy and needs to be assessed and evaluated as to whether it meets national and international standards. Long term funding needs to be secured.

Ways need to be sought to retain COM graduates in the country. Similarly conditions must improve for those who are currently abroad and who have finished training, but who are reluctant to come back mainly for financial reasons. Most important are:

1. An improved and realistic pay package is needed that ensures housing, transport and school fees for children. Salary supplementation may come through research programmes, donor support or private practice.

2. There is a need for a structure for adequate career development. This is largely in place. The COM has an excellent academic climate with continuous interactions with international experts and research units that collaborate in research and teaching. Career opportunities are abundant, as are opportunities for further training or specialization. There are numerous vacancies that currently cannot be filled.

3. Students at the COM should be stakeholders in their own future. At present their training is heavily subsidized by government and they only pay a nominal fee. Investment by Government should imply an obligation for a period of service after graduation.

4. There is a need for provision of opportunities for research. The College is uniquely placed for developing research programmes with its partners such as the Malawi-Liverpool-Wellcome link and the Johns Hopkins University, which could provide attractive career perspectives.

5. There is a need for national representation. As the COM is a training institution, it is responsive to the needs of the Ministry of Health (MoH) as a major stakeholder. For those who do not pursue a career in academic medicine, career perspectives within the MoH should be clear with regard to job opportunities and remuneration after training in the COM.

## Conclusion

The pool of Malawian doctors is still small but there seems to be a trend that graduates remain in the country. The postgraduate programme is in place to train graduates to become senior staff members themselves. For the interim period, it is essential that donors are convinced that technical assistance at the highest academic levels will be necessary for the foreseeable future, in order to ensure that quality of teaching is not compromised and that the postgraduate programme is successful. Only then will we see a successful transition to a pre- and postgraduate training programme of high standard, wholly owned and administered by well trained Malawian professionals.

## Competing interests

The author(s) declare that they have no competing interests.

## Authors' contributions

EEZ collected and analysed the data and drafted the paper

RLB critically revised the data and helped in drafting the paper

Both authors have read and approved the manuscript.

## References

[B1] Core health Indicators. http://www.WHO.int/whosis.

[B2] Broadhead RL, Muula AS (2002). Creating a medical school for Malawi: problems and achievements. BMJ.

[B3] Eastwood JB, Conroy RE, Naicker S, West PA, Tutt RC, Plange-Rhule J (2005). Loss of health professionals from sub-Saharan Africa: the pivotal role of the UK. Lancet.

[B4] Saravia NG, Miranda JF (2004). Plumbing the brain drain. Bull World Health Org.

[B5] Hagopian A, Thompson MJ, Fordyce M, Johnson KE, Hart LG (2004). The migration of physicians from sub-Saharan Africa to the United States of America: measures of the African brain drain. Human Resources for Health.

[B6] Dambisya YM (2004). The fate and career destinations of doctors who qualified at Uganda's Makerere Medical School in 1984: retrospective cohort study. BMJ.

[B7] Martey JO, Hudson CN (1999). Training specialists in the developing world: ten years on, a success story for West Africa. Br J Obst Gyn.

[B8] WHO 2005 University of Malawi: College of Medicine external evaluation report: 1–5 August 2005.

